# Lack of knowledge about the hypotensive effects of potassium and dairy: current hypertension-related knowledge and results of a knowledge intervention in Japanese workers

**DOI:** 10.1539/eohp.2025-0026

**Published:** 2025-12-19

**Authors:** Aya Higashiyama, Nagako Okuda, Kyoko Kojima, Yuki Yonekura, Kozo Tanno, Akira Okayama

**Affiliations:** 1 Department of Hygiene, Wakayama Medical University, Wakayama, Japan; 2 Division of Applied Life Sciences, Graduate School of Life and Environmental Sciences, Kyoto Prefectural University, Kyoto, Japan; 3 The Research Institute of Strategy for Prevention, Tokyo, Japan; 4 Department of Nursing Informatics, Graduate School of Nursing Science, St. Luke’s International University, Tokyo, Japan; 5 Department of Hygiene and Preventive Medicine, Iwate Medical University, Yahaba, Japan

**Keywords:** dairy products, hypertension, knowledge, population, primary prevention

## Abstract

**Objectives:**

A high dietary sodium/potassium ratio with low potassium and high sodium intake, which is a risk factor for hypertension, is a characteristic of diets in East Asia, such as Japan. To promote prevention of hypertension among Japanese workers, knowledge of the antihypertensive effects of potassium, which is not limited to vegetables and fruits and include various potassium-rich foods, such as milk, is important. Thus, we investigated the knowledge of workers on the associations of potassium and milk intake with blood pressure and effective methods for communicating this association.

**Methods:**

The participants were 130 Japanese workers, most of whom were under 50 years old and were not using medication for hypertension. After distributing only free dairy products for 3 weeks, leaflets and stickers were distributed with the dairy products to communicate the knowledge about the hypotensive effect of potassium and milk for 3 weeks. At baseline, after distributing only free dairy products, and after providing knowledge, the participants responded to the same questionnaires on the association of potassium and milk intake with blood pressure. Questionnaire responses were analyzed using Cochran’s Q test and multiple comparison analysis.

**Results:**

The percentage of the correct answers on the association of potassium and milk intake significantly improved with the knowledge intervention (35.4% at baseline, 50.8% after distributing only the dairy products, and 90.0% after distributing the knowledge regarding potassium).

**Conclusions:**

The participants had limited knowledge about the association between potassium and milk intake and blood pressure. Our intervention methods might be effective for improving knowledge of the association.

## Introduction

Hypertension (HT) is one of the most important risk factors of cardiovascular diseases (CVDs)^1,2)^ and remains the leading cause of death worldwide^3)^. The prevalence of HT is approximately 5% among Japanese individuals aged 20–39 years but increases to over 50% among those aged 60–69 years^4)^. Therefore, early prevention, including knowledge provision from a young age among the working population, is important to decrease the overall HT prevalence in the population.

The diets in East Asian countries, such as China and Japan, are characterized by high sodium (Na) and low potassium (K) intake, which is a major modifiable risk factor for HT^2,5,6,7)^. In the Japanese population, the mean daily salt intake has gradually decreased; however, it has remained at approximately 10 g, with no apparent downward trend in recent years^8)^. On the other hand, the mean daily K intake is approximately 2,200 mg^8)^, which is much lower than 3,500 mg recommended by the World Health Organization^9)^. Previous studies have demonstrated that higher urinary Na/K ratio is associated with higher BP, and higher dietary Na/K ratio is associated with higher CVD mortality^10,11)^. Recently, a large cluster-randomized controlled trial (RCT) in China showed that increasing the intake of K can serve as an important population-level intervention for the reduction of CVD events, particularly in populations with high Na and low K intake^6,12)^. In this RCT, a significant reduction in CVD events and mortality was reported with reduced Na and increased K excretion in the urine by replacing regular salt with a K-enriched salt substitute^12)^.

Although fruits and vegetables have often been encouraged to consume in many health education settings to prevent HT, it is not clear whether individuals know why these foods are recommended. Understanding the relationship between K intake and blood pressure (BP) might encourage individuals to consume more vegetables, fruits, and other variety of K-rich foods. A few studies have investigated knowledge about the hypotensive effect of fruits and vegetables intake^13,14)^; however, our previous study was the only one that investigated knowledge about the hypotensive effect of K^15)^. In this study conducted among Japanese workers, only 35% of the participants knew the BP-lowering effect of K, whereas 98% and 80% knew the effects of reducing salt and the intake of fruits and vegetables, respectively^15)^.

In addition to fruits and vegetables, another K-rich food is milk. Although the hypotensive efficacy of milk has been reported^16,17,18,19)^ and the addition of milk (200 g) is effective for reducing the dietary Na/K ratio per meal^20)^, few studies have investigated knowledge about the association of milk intake with BP among the population. Considering that the daily intake of dairy products (approximately 110 g in 2023) of Japanese adults^8)^ is lower than the recommended level (200 g)^21)^, the consumption of target amounts of milk should be encouraged in the Japanese population. Thus, among Japanese workers, we performed a study to investigate the current state of HT-related knowledge, such as diseases for which HT is a risk factor and the associations between BP and lifestyles, including K and milk intake. We also performed an intervention to increase that knowledge.

## Methods

### Study participants

This study was conducted from the beginning of April 2023 to the end of May 2023 in a company cafeteria located within a manufacturing company in Osaka Prefecture, Japan. The company employs approximately 400 employees (300 men and 100 women). Among them, 60 are engaged in administrative services, 220 in technology and development, 70 in production management, and 50 in factory operations. A food service company offers employees several lunch menus daily, but some employees bring their own lunch and eat at the cafeteria. Thus, cafeteria users were defined as employees who ate lunch menus provided by the food service company and those who brought their own lunch to the cafeteria.

The number of regular cafeteria users per day was approximately 200. A total of 162 individuals responded to the questionnaires, which were administered three times during the research period, and 130 individuals who completed all three questionnaires were included in this study. Given that we planned to perform the intervention by distributing free dairy products, an employee who had an estimated glomerular filtration rate of <45 mL/min/1.73 m^2^ was excluded from the study recruitment to avoid the adverse effects of increased K intake from dairy.

Written informed consent was obtained from all participants. This study was conducted according to the Declaration of Helsinki and the Ethical Guidelines for Medical and Biological Research Involving Human Subjects (revised March 10, 2022). All procedures involving the participants were approved by the Ethics Review Board of Wakayama Medical University (No. 3554). This study has been registered at UMIN along with an intervention study to improve BP by using low-Na/K seasonings and adding dairy to lunch menu in work places (UMIN000050876).

### Baseline survey about HT-related knowledge

**Fig. 1.**
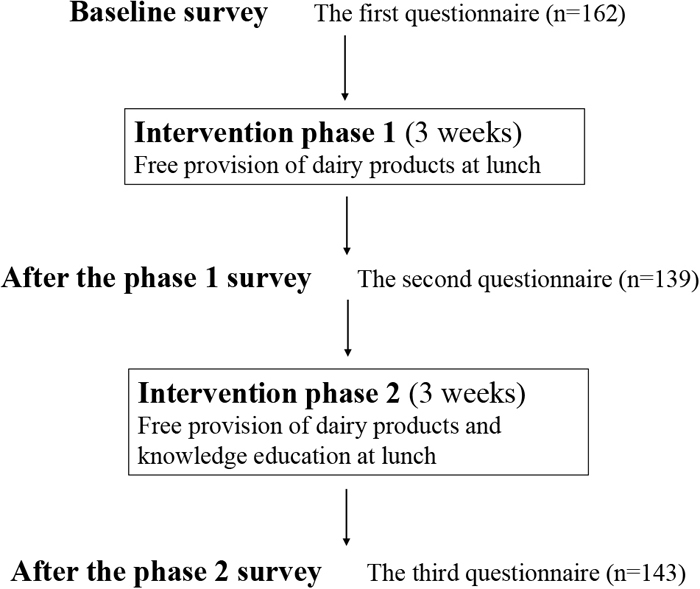
The flow of the study.

Figure 1 shows an overview of this study. In the baseline survey, participants were requested to respond to a questionnaire about their characteristics and HT-related knowledge, such as diseases for which HT is a risk factor and the association of lifestyle with BP. The questionnaire was used three times in total, with same questions; one for baseline survey and twice in the intervention period. eTable 1 provides the details of the questionnaires used in this study.

### Knowledge intervention

After the baseline survey, the intervention, which consists of two phases, was performed (Figure 1). In phase one, to observe changes in the knowledge by provision of dairy products only, several dairy products were distributed free of charge for 3 weeks. Individual single-serving-size products, such as milk (200 mL), low-fat milk (200 mL), yogurt (112 g), yogurt with various fruit flavors (200 g), and milk beverages (112 g), were provided to the participants. At the end of phase one, the second questionnaire was administered with contents that were similar to the baseline survey.

After collecting the responses to the second questionnaire, phase two was performed. By adopting the same free distribution of dairy products as in phase one, a knowledge intervention was performed using leaflets and stickers to communicate HT-related knowledge at lunchtime in the cafeteria. Eleven leaflets and eight stickers were prepared for the knowledge intervention. Each leaflet was 14.8 cm in width and 10.5 cm in height; contained a message that was focused on one important piece of knowledge, such as “milk is rich in K and poor in Na (salt)”; and had friendly illustrations for the public. Each sticker was 7.3 cm in width and 2.5 cm in height and contained a short summary of the message of the corresponding leaflet and a tune that could be sung with rhythms (called “senryu verse” in Japanese) and was attached to a dairy product. eTable 2 shows a summary of the contents of all leaflets. Each leaflet also contained the following message: “if you are instructed to limit K intake, please discuss with your doctor about the leaflets”.

One leaflet per day was distributed in order presented in eTable 2; given that phase two was conducted over 15 workdays, leaflet numbers 3–6 were distributed twice during this period. The other leaflets were distributed only once during the study period. The research staff, all of whom were dietitians, distributed the leaflets and the dairy products with stickers at the cafeteria. After phase two, the third questionnaire, which had contents that were similar to the baseline survey and included questions about the assessment of the intervention methods, was administered.

### Statistical analyses

The distribution of the frequency of free dairy product consumption per week per person was compared between phases one and two using the Wilcoxon signed-rank test. The responses to the questionnaires about knowledge (correct or incorrect) were compared at baseline, after phase one, and after phase two using Cochran’s Q test with multiple comparisons (Bonferroni correction). The significance level was set at p<0.05 (two sided). All statistical analyses were performed using SPSS version 26 (IBM Japan Ltd., Tokyo, Japan).

## Results

Among 130 participants, 86 individuals (66.2%) were men, and 102 individuals (78.5%) were aged under 50 years old (Table 1). Regarding the daily operations of the participants, the percentage of technology and development participants (38.5%) was lower than that of all employees (55%). The percentage of participants who were told that their BP was high was 25.4%, and that of participants taking medication for HT was 4.6%.

**Table 1.** Characteristics of the participants

		Number	(%)
Age, years			
	<30	29	(22.3)
	30–39	39	(30.0)
	40–49	34	(26.2)
	50–59	23	(17.7)
	≥60	4	(3.1)
	Unknown	1	(0.8)
Sex			
	Men	86	(66.2)
Daily operations			
	Administrative services	25	(19.2)
	Technology and development	50	(38.5)
	Production management	25	(19.2)
	Factory operations	22	(16.9)
	Other	7	(5.4)
	Administrative services, technology, and development	1	(0.8)
	Previous high blood pressure noted	33	(25.4)
	Present medication for hypertension	6	(4.6)

Table 2 shows the number of free dairy products consumed per week in phases one and two. The distribution was not significantly different before and after the knowledge intervention. In phase one, a small number of participants did not wish to receive free dairy products owing to their dislike of milk; however, they wished to receive the leaflets, and some of them obtained dairy products, such as yogurt, in phase two.

**Table 2.** Number of users of free dairy products according to the number consumed per week before and after knowledge intervention

	Number of times per week							
	0	<1	1	2	3	4	5	≥6
During the free distribution of dairy products only (in phase 1)	4	3	8	8	31	22	53	1
During the distribution of free dairy products and knowledge intervention (in phase 2)	1	2	16	12	20	23	50	6
								*p*-value=0.857

*p*-value: Wilcoxon signed-rank test for the difference between the distribution after phases 1 and 2.

Table 3 and Table 4 show the responses to the questionnaires about knowledge at baseline, after phase one, and after phase two. Regarding the association of HT with disease risk (Table 3), most participants correctly identified the associations even at baseline, except in relation to kidney disease. Regarding the risk of HT for stroke, myocardial infarction, and dementia, the percentage of correct answers improved after phases one and two. Regarding the association of milk consumption with disease risk (Table 3), more than 90% of the participants knew the preventive effect of milk against osteoporosis at baseline. However, the antihypertensive effect of milk was not well recognized at baseline or after phase one, and the knowledge intervention significantly increased the percentage of correct responses. For the association of milk with the risk of stroke and myocardial infarction, after phase two, more than 60% of the participants responded that milk is associated with decreased risk for stroke and myocardial infarction. For the association of milk with the risk of diabetes and dyslipidemia, the *p*-value for Bonferroni’s correction was significant after the knowledge intervention; however, the percentage of correct answers was still low after phase two. Regarding the substances in milk (Table 3), more than 90% of the participants knew that calcium is a component of milk. However, the percentage of participants who knew that milk contains K was approximately 30–40% at baseline and after phase one; this increased to 82%, with statistical significance, after phases two.

**Table 3.** Response to the questionnaires about knowledge at baseline, after the provision of dairies only, and after knowledge intervention plus provision of dairy products

		Number and percentage of participants who answered “Yes”								
		Baseline		After phase 1			After phase 2			p*
				(After the provision of dairy products only)			(After the provision of dairy products and knowledge intervention)			
		N	(%)	N	(%)	*p*† between baseline and after phase 1	N	(%)	*p*† between after phases 1 and 2	
Hypertension increases the risk for the follwing diseases:										
	Stroke	108	83.1	124	95.4	<0.001	123	94.6	n.s.	<0.001
	Myocardial infarction	99	76.2	115	88.5	<0.01	120	92.3	n.s.	<0.001
	Dementia	19	14.6	32	24.6	<0.05	40	30.8	n.s.	<0.001
	Stomach cancer	11	8.5	15	11.5	n.s.	22	16.9	n.s.	<0.05
	Kidney diseases	28	21.5	37	28.5	n.s.	46	35.4	n.s.	<0.01
Milk intake decreases the risk of the following:										
	Osteoporosis	120	92.3	124	95.4	n.s.	122	93.8	n.s.	n.s.
	Hypertension	33	25.4	49	37.7	n.s.	96	73.8	<0.001	<0.001
	Diabetes	16	12.3	21	16.2	n.s.	44	33.8	<0.001	<0.001
	Dyslipidemia	23	17.7	31	23.8	n.s.	57	43.8	<0.001	<0.001
	Myocardial infarction	23	17.7	33	25.4	n.s.	85	65.4	<0.001	<0.001
	Stroke	21	16.2	33	25.4	n.s.	82	63.1	<0.001	<0.001
The following are the main substances of milk:										
	Calcium	124	95.4	126	96.9	n.s.	122	93.8	n.s.	n.s.
	Sodium	25	19.2	36	27.7	n.s.	42	32.3	n.s.	<0.05
	Potassium	40	30.8	53	40.8	n.s.	107	82.3	<0.001	<0.001
	Iron	40	30.8	54	41.5	<.05	50	38.5	n.s.	<0.05
	Zinc	28	21.5	26	20.0	n.s.	37	28.5	n.s.	n.s.

p*: *p*-value value for Cochran’s Q test. *p*†: *p*-value for Bonferroni correction.

**Table 4.** Response to the questionnaires about knowledge at baseline, after the provision of dairies only, and after knowledge intervention plus the provision of dairy products

		Correct answer	Number and percentage of participants answered correctly								
			Baseline		After phase 1			After phase 2			p*
					(Aftern provision of dairy products only)			(Aftern provision of dairy products and knowledge intervention)			
			N	(%)	N	(%)	*p*† between baseline and after phase 1	N	(%)	*p*† between after phases 1 and 2	
The following lifestyles make blood pressure increase or decrease:											
	Salt intake	increase	121	93.1	128	98.5	n.s.	126	96.9	n.s.	n.s.
	Potassium intake	decrease	46	35.4	66	50.8	<0.05	117	90.0	<0.001	<0.001
	Fruits and vegetable intake	decrease	108	83.1	121	93.1	<0.05	124	95.4	n.s.	<0.001
	Exercise	decrease	113	86.9	117	90.0	n.s.	116	89.2	n.s.	n.s.
	Obesity	increase	123	94.6	126	96.9	n.s.	128	98.5	n.s.	n.s.
	Alcohol drinking 2 gou (4 units) and more/day	increaase	111	85.4	125	96.2	<0.01	125	96.2	n.s.	<0.001
	Emotional stress	increase	117	90.0	123	94.6	n.s.	126	96.9	n.s.	<0.05
	Smoking	increase	112	86.2	117	90.0	n.s.	122	93.8	n.s.	<0.05

p*: *p*-value value for Cochran’s Q test. *p*†: *p*-value for Bonferroni correction.

As shown in Table 4, the association of lifestyle with BP was well recognized even at baseline, except for K intake. The percentage of correct answers on the effect of K on BP was 35.4% at baseline, 50.8% after phase one, and 90.0% after phase two, with statistical significance.

In the questionnaire after phase two, the participants responded to the questions about the frequency of reading the leaflets and the stickers and the ease of understanding the contents of those education media as follows (leaflets versus stickers): “read almost every time” (74.6% vs. 65.4%) and “read two in three times” (10.0% vs. 11.5%), “understood the contents very well” (64.6% vs. 55.4%) and “understood the contents approximately” (27.7% vs. 30.8%) (eTable 3).

**Fig. 2.**
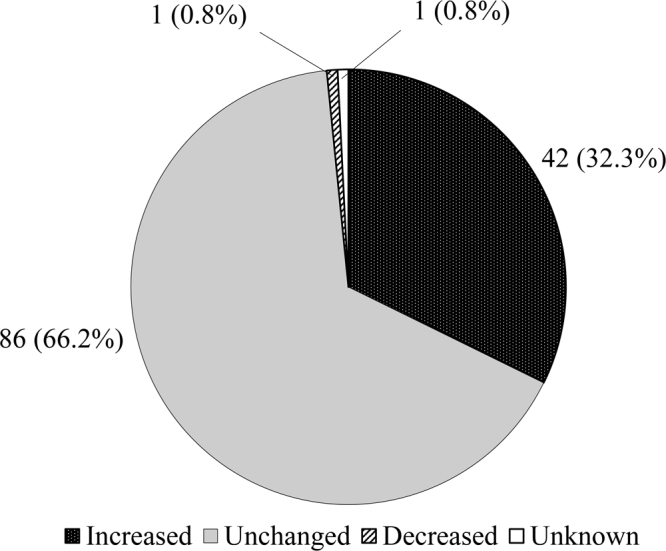
Distribution of the participants according to the change in the consumption of dairy products at home after knowledge intervention compared with those at baseline.

Figure 2 shows the distribution of the participants according to the change in the consumption frequency of dairy products at home after the knowledge intervention compared with that at baseline. Approximately 30% of the participants self-reported that their consumption frequency increased during the study.

## Discussion

The Japanese workers of this study, most of whom were <50 years old and were free from medication for HT, were mostly familiar with the increased risk of HT due to salt intake and the hypotensive effect of intake of fruits and vegetables; however, they were not familiar with the hypotensive effect of K and milk intake. After the intervention, which involved the provision of information on the hypotensive effect of K intake and milk consumption by short messages, the participants’ knowledge significantly improved. In addition, approximately 30% of the participants self-reported that the frequency of dairy consumption at home increased after the knowledge intervention compared with that in the baseline.

As the prevalence of raised BP and adverse impact of HT on CVD continue to increase globally^1)^, studies on HT-related knowledge have been performed in the general population of many countries_since the 1970s^13,14,15,22,23,24,25,26,27,28)^. Although many of those studies have investigated knowledge of the association of salt intake with BP, only a few have investigated knowledge of the association of vegetables/fruits^13,14)^ and K intake^15)^ with BP.

Regarding interventions for HT-related knowledge in the general population, only a few studies that investigated BP changes after lifestyle interventions reported improvement in knowledge^29)^. A cluster RCT among Chinese community dwellers at high risk for HT^29)^ showed that an integrative program including health education, physician follow-up, and self-management significantly improved HT-related knowledge and reduced the incidence of HT in the intervention groups compared with the control groups. However, diet education in the trial was limited to salt reduction. Therefore, to the best of our knowledge, the present study is the first to investigate the effect of an intervention providing knowledge about the association of K intake with HT and K source foods, including dairy products. As a result of the intervention, participants’ knowledge about the hypotensive effects of K and dairy improved.

The uniqueness of this study lies in the possibility of easily applying its intervention methods in the real world. This method could be also applicable to population strategies targeting community dwellers in commercial facilities (ie, supermarkets) or health promotion events.

In Japan, information on lifestyle changes for CVD prevention has been provided to all workers and community residents who undergo annual specific health checkups and specific health guidance since 2008^30)^. Therefore, the association of lifestyle with HT is considered well known to the Japanese population. At the baseline of the present study, the increased risk of HT due to salt intake, which is commonly provided in health guidance, was well known. However, the hypotensive effect of K was not recognized, whereas that of fruit and vegetable intake was well known. These results suggest that the participants knew about the association of vegetables and fruits with BP but did not know the reason. In populations with low K intake, such as the Japanese, HT-related knowledge that is not biased toward salt reduction is needed to effectively reduce dietary Na/K ratios. To increase total K intake, it will be difficult for individuals to achieve the recommended K intake by increasing vegetables and fruits intake alone without keys of dietary instruction; that is, the association of K with BP and various K-rich foods that are readily available in individuals’ diet culture, while urging caution for individuals at high risk of hyperkalemia. Introducing milk and yogurt as one of the K-rich foods may provide individuals with more opportunities to consume various K-rich foods in addition to fruits and vegetables.

Although the BP-lowering effects of a diet rich in vegetables, fruits, and dairy products and poor in saturated fatty acids and cholesterol have already been demonstrated^31,32,33)^, the K-related antihypertensive effect of milk consumption was unknown at baseline of the present study. In populations where the mean intake of dairy products has not reached the recommended amount, dairy products that are easy to consume could be introduced as one of the K-rich foods. Furthermore, in Japan, the consumption of milk and other dairy products among individuals ≥20 years (110.7 g for milk and other dairy products and 61.9 g for milk only^4)^) is below the recommended amount (200 g/day for milk)^21)^. Accordingly, the consumption of dairy products, such as a cup of milk (200 g) per day, would be recommended, particularly low-fat products^18,34)^, to prevent an increase in blood cholesterol levels.

In this study, the following pieces of information were not well known to the participants: 1) K has a BP-lowering effect; 2) K is one of the ingredients in milk; and 3) the association between milk consumption and BP. These pieces of information were generally well communicated after the knowledge intervention. In phase one, we first observed changes in knowledge due to factors other than the leaflets and stickers. The dairy products did not contain any information on health effects, which we provided in phase two. Furthermore, the association of K with BP was not advertised in public media throughout the study period. The most likely reason for the improvement in knowledge about the association of K with BP after phase one was that, after responding to the baseline questionnaire, some participants reviewed or recalled a big leaflet that the medical insurer had provided them in the past. The medical insurer provided information on the association of fruits and vegetables intake and alcohol consumption with BP. The leaflet distributed by the medical insurer also stated in a small print that K in fruits and vegetables lowers BP; however, this information was probably not noticeable because there was a lot of other information, such as salt reduction or exercise. Therefore, while some of the *p*-values presented in Table 4 showed significant differences between baseline and after phase one, the corresponding percentage in the association of K with BP did not improve sufficiently. On the other hand, after phase two compared to after phase one, the effectiveness of the knowledge intervention was clear by the significant *p*-value and the prominent improvement in the percentage of correct answers about the association of K and milk with BP and about the ingredients of milk.

The leaflets and stickers in this study were characterized by the clarity of their respective messages. Each leaflet consisted of one message with short texts and a few illustrations reflecting the message, and the stickers contained only a short sentence that further summarizes each leaflet’s message. If a participant had seen a sticker without reading the corresponding leaflet, it might have been difficult for them to understand the message. However, according to the responses of the participants after phase two, both their understanding of the content and the number of times the medium was read were generally good. Therefore, a small leaflet and sticker could be effective methods for communicating the benefits of K and dairy products for BP in a population strategy. Owing to the development of the internet, virtual interventions are expected to increase in the future. The method used in this study to provide knowledge in as limited a manner as possible can be applied to Internet-based interventions. However, to communicate information widely using the population approach, it might be difficult to assume that the target population would make an effort to obtain information on their own. According to the participants’ responses after phase two, the most likely means of knowledge transfer were hand-out leaflets (30.8%), followed by stickers (21.5%), leaflets attached to dairy products (19.2%), posters on the sales floor (13.1%), and Internet-based media (15.4%). Furthermore, in a previous study of Australian general practitioners, the top preference for accessing information related to BP management was a one-page summary of specific topics, followed by a website^35)^. Therefore, there might still be a demand for leaflets in which the content can be understood at a glance to communicate information. Knowledge should be communicated by selecting the best means that are likely to be read by individuals on the basis of the purpose and target populations.

In this study, there might have been a change in practice among the participants. More than 30% of the participants responded that dairy consumption at home increased at the end of the study compared with at the baseline. Given that the message of the intervention was to “increase” milk/yogurt intake, the acceptability of the knowledge to the practice might be different from that of salt or alcohol “reduction.” A previous intervention study in China reported that the acquisition of knowledge about the association of salt intake with BP did not significantly affect behavior; similar results were observed for alcohol consumption^29)^. These discrepancies between knowledge acquisition and practice are likely due to fact that the pathways between knowledge and behavioral change could be influenced by many other factors, such as difficulty in changing habits or lack of environmental support^29)^. Furthermore, it is usually more acceptable to develop new behaviors than to change or abandon existing behaviors^29)^. Therefore, having a cup of milk or yogurt per day could be an easier lifestyle change than reducing salt intake because of the following reasons: 1) individuals are not influenced by family dietary preferences; 2) milk and yogurt are easy to purchase; and 3) individuals can consume them without cooking. However, as is true for almost all lifestyle modifications related to diet, it is necessary to instruct individuals to consume adequate amounts of dairy products within the recommended levels. Furthermore, it is necessary to increase the accessibility of dairy products in daily life, such as the price and availability of these products in the workplace, and to improve the taste of low-fat products.

The knowledge intervention significantly increased correct response rates regarding the association of intakes of K and dairy with HT, as well as between dairy intake and stroke. However, the proportion of respondents who answered affirmatively regarding the association between dairy products and dyslipidemia or myocardial infarction that had not been included in the leaflets and stickers also increased by 20–30%. This increase suggests the presence of the effect of administering the same questionnaires repeatedly. This is a significant limitation of single-arm trials, and the effectiveness of knowledge interventions using similar methods requires further verification in future intervention studies with separately established control group.

This study has several other limitations. First, changes in the frequency of dairy product consumption at home were self-reported and not evaluated using objective measures. Second, it is unknown whether the knowledge acquired has been retained and whether the participants who reported an increase in dairy consumption at home still consume more dairy products than before the study. Third, the representativeness of the results in this study on the general Japanese population is unknown because there are few reports on public HT-related knowledge in Japan. Finally, whether knowledge acquisition prevented HT or improved BP in the population was not investigated.

In conclusion, this study showed that young to middle-aged Japanese workers, most of whom were free from HT medication, did not have adequate knowledge of the association of K and milk intake with BP. The knowledge of the participants was significantly improved using small print media, which minimized the message that need to be communicated. Furthermore, the possibility of increased dairy consumption was suggested by approximately 30% of the participants through the intervention. In populations with low K and dairy intakes, such as the Japanese, it is important to disseminate knowledge of the association of K with BP and various K-rich foods, along with a reduction in salt intake, to lower the dietary Na/K ratio. The knowledge intervention conducted in this study may be effective in increasing knowledge about K in population strategies.

## Supplementary Material

Supplementary eTable 1

Supplementary eTable 2

Supplementary eTable 3

## Data Availability

The data are not available for public access. Further inquiries can be directed to the corresponding author.
