# Supplementary material for: Lack of knowledge about the hypotensive effects of potassium and dairy: current hypertension-related knowledge and results of a knowledge intervention in Japanese workers
Source: Environ Occup Health Pract. 2025 Dec 19;8(1):2025-0026. doi: 10.1539/eohp.2025-0026 (PMC13012896; doi:10.1539/eohp.2025-0026)
Supplement: Supplementary file 1 — Supplementary eTable 1 [file eohp-8-2025-0026-s001.pdf]

eTable 1. Questions and options of the response in the questionnaires used in the study

Question	Options of response
Hypertension and the risk of disease	
The risk of the following diseases are increased among hypertensives.	Stroke
Choose the correct ones as many as you like.	Myocardial infarction
	Dementia
	Stomach cancer
	Renal diseases
Lifestyles and blood pressure	
Indicate the association of each lifestyle and blood pressure.	
Increased salt intake	Increase, Decrease, Unknown
Increased potassium intake	Increase, Decrease, Unknown
Increased fruit and vegetable intake	Increase, Decrease, Unknown
Regular exercise	Increase, Decrease, Unknown
Obesity	Increase, Decrease, Unknown
Drinking alcohol 2 gou (4 drinks)/day and more	Increase, Decrease, Unknown
Persistent mental stress	Increase, Decrease, Unknown
Smoking	Increase, Decrease, Unknown
Milk consumption and the risk of the disease.	
The risk of the following diseases are decreased among individuals who drink milk regulary.	Osteoporosis
Choose the correct ones as many as you like.	Hypertension
	Diabetes Mellitus
	Dyslipidemia (hypercholesterolemia)
	Myocardial infarction
	Stroke
Ingredients of milk	
Milk is rich in the following minerals.	Calcium
Choose the correct ones as many as you like.	Sodium
	Potassium
	Iron
	Zinc
Number of times free dairy products taken	
Identify the times you take the free dairy products provided in the recent three weeks.	times per week
Assessment of the leaflets	
Choose the times you read the leaflets provided from the options.	Read them almost every time, Read them two in three times, Read them one in three times, Almost did not read
Choose the option about the ease of understanding the contents of the leaflets	Understood the contents very well, Understood the contents approximately,<br>Hardly understood
Assessment of the stickers	
Choose the times you read the stickers provided from the options.	Read them almost every time, Read them two in three times, Read them one in three times, Almost did not read
Choose the option about the ease of understanding the contents of the stickers	Understood the contents very well, Understood the contents approximately,<br>Hardly understood
Change in the frequency of dairy products consumption before and after the study	
Choose the option about the change of dairy products consumption at home after the study compared to before the study.	Increased, Unchanged, Decreased
