# Supplementary material for: Lack of knowledge about the hypotensive effects of potassium and dairy: current hypertension-related knowledge and results of a knowledge intervention in Japanese workers
Source: Environ Occup Health Pract. 2025 Dec 19;8(1):2025-0026. doi: 10.1539/eohp.2025-0026 (PMC13012896; doi:10.1539/eohp.2025-0026)
Supplement: Supplementary file 2 — Supplementary eTable 2 [file eohp-8-2025-0026-s002.pdf]

eTable 2. Main messages on each leaflet used in the knowledge intervention

Leaflet number	Message presented in the leaflet
1	Let's start lifestyle modification before you start having lifestyle-related diseases.
2	Potassium in fruits and vegetables lower your blood pressure.
3	Salt intake increases your blood pressure. Potassium intake lowers your blood pressure.
4	Milk and yogurt are poor in salt and rich in potassium and calcium. Potassium and calcium in milk can help lower BP.
5	Potassium intake is lower than the recommended level in the Japanese population.
6	An epidemiological study in Japan suggested that a cup of milk/day might prevent cerebral infarction [36)].
7	Tofu and Natto (Japanese soy products), vegetable juices, and fruits are also rich in potassium.
8	Change your snacks from chocolates or salty Japanese rice crackers to fruits.
9	You can reduce salt intake by using "Nutrition Facts Label" (check the number displayed as salt equivalent).
10	You can increase your potassium intake by adding the diplayed dishes.
11	Potassium rich foods, such as vegetables and soy products, are rich in diet fibers and vegetable proteins with low energies.

In each leaflet, the following message was shown: "If you are instructed to limit potassium intake, please discuss with your doctor about the leaflets."
One leaflet per day was distributed, as indicated by the number presented; given that phase 2 was conducted over 15 workdays during a 3-week period, leaflet numbers 3–6 were distributed twice during the period. The other numbered leaflets were distributed once during the period.
