# Supplementary material for: Lack of knowledge about the hypotensive effects of potassium and dairy: current hypertension-related knowledge and results of a knowledge intervention in Japanese workers
Source: Environ Occup Health Pract. 2025 Dec 19;8(1):2025-0026. doi: 10.1539/eohp.2025-0026 (PMC13012896; doi:10.1539/eohp.2025-0026)
Supplement: Supplementary file 3 — Supplementary eTable 3 [file eohp-8-2025-0026-s003.pdf]

eTable 3. Response of the participants to the leaflets and the stickeres after phase 2

	Leaflets		Stickers	
	n	%	n	%
Frequency of reading				
Almost every time	97	74.6	85	65.4
Two in three times	13	10.0	15	11.5
One in three times	7	5.4	10	7.7
Almost did not read	13	10.0	20	15.4
Ease of understanding the contents				
Understood the contents very well	84	64.6	72	55.4
Understood the contents approximately	36	27.7	40	30.8
Hardly understood	7	5.4	14	10.8
No response	3	2.3	4	3.1
